# Towards OCT-Guided Endoscopic Laser Surgery—A Review

**DOI:** 10.3390/diagnostics13040677

**Published:** 2023-02-11

**Authors:** Ajay Gunalan, Leonardo S. Mattos

**Affiliations:** 1 Department of Advanced Robotics, Istituto Italiano di Tecnologia, 16163 Genoa, Italy; 2 Department of Informatics, Bioengineering, Robotics and Systems Engineering, University of Genoa, 16145 Genoa, Italy

**Keywords:** endoscopy, OCT, laser surgery, theranostics systems

## Abstract

Optical Coherence Tomography (OCT) is an optical imaging technology occupying a unique position in the resolution vs. imaging depth spectrum. It is already well established in the field of ophthalmology, and its application in other fields of medicine is growing. This is motivated by the fact that OCT is a real-time sensing technology with high sensitivity to precancerous lesions in epithelial tissues, which can be exploited to provide valuable information to clinicians. In the prospective case of OCT-guided endoscopic laser surgery, these real-time data will be used to assist surgeons in challenging endoscopic procedures in which high-power lasers are used to eradicate diseases. The combination of OCT and laser is expected to enhance the detection of tumors, the identification of tumor margins, and ensure total disease eradication while avoiding damage to healthy tissue and critical anatomical structures. Therefore, OCT-guided endoscopic laser surgery is an important nascent research area. This paper aims to contribute to this field with a comprehensive review of state-of-the-art technologies that may be exploited as the building blocks for achieving such a system. The paper begins with a review of the principles and technical details of endoscopic OCT, highlighting challenges and proposed solutions. Then, once the state of the art of the base imaging technology is outlined, the new OCT-guided endoscopic laser surgery frontier is reviewed. Finally, the paper concludes with a discussion on the constraints, benefits and open challenges associated with this new type of surgical technology.

## 1. Introduction

Over 80% of all cancers are attributed to epithelial tissue, and their early-stage detection can substantially reduce the death rate [1]. However, conventional biopsy to detect cancer has some disadvantages, such as invasiveness, sampling error, time consumption, processing artifacts, and interpretative variability. In addition, the identification of the suspected tissue volume is challenging as precancerous lesions do not show significant color changes or morphological differences compared to the healthy ones under white light illumination [2]. OCT has a good potential to address these challenges given the following: (1) its imaging resolution ranges between 5 and 10 μm, which is enough to resolve precancerous lesions; (2) its penetration depth is normally between 1 and 3 mm, which is sufficient to image the entire depth of epithelial tissues; and (3) it is an optical fiber-based imaging system and thus has potential to be compact, free of radiation, and affordable [3].

Optical Coherence Tomography (OCT) is a non-invasive imaging technique that resembles ultrasound imaging. However, unlike ultrasound, it uses an infrared laser to image both surface and subsurface details. OCT images are fundamentally made up of a one-dimensional point scan called an A-scan. A point on the sample is scanned to acquire its raw spectral data, consisting of spectrum intensity along various depths. These data are processed to obtain the A-scan data, which represents the reflectivity signal strength at various depths in the direction of that point. When the A-scan is repeated along a line in the sample, we obtain a B-scan (2D images). When the B-scan is repeated along the surface of the sample, we obtain a C-scan (3D images). Laser scanners perform the B-scan and C-scan. These laser scanners can be based on different technologies, such as mirror galvanometers, MEMS-mirrors, MEMS-lenses or fiber scanners.

The most straightforward OCT configuration is based upon the Michelson interferometer, which splits the light 50/50 into a sample arm and a reference arm. In this simple configuration, nearly 75% of the optical power is wasted [4]. The SNR of OCT is approximately proportional to the optical power and is inversely proportional to the bandwidth of the detection electronics. Hence, alternative power-conserving configurations have been developed. As described in [4], such alternative configurations may use optical circulators, unbalanced couplers, and/or balanced heterodyne detection.

**Figure 1:**
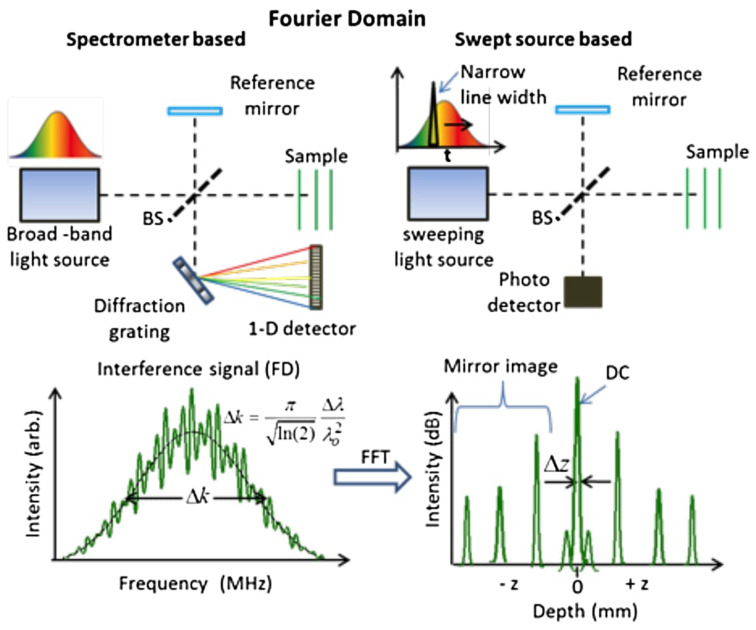
Comparison between Spectral Domain OCT (SD-OCT) and Swept Source OCT (SS-OCT) by [9]. Figure licensed under CC BY 4.0.

The first-generation OCT is known as Time-Domain OCT (TD-OCT). In these systems, the reference arm was mechanically scanned, limiting the image acquisition rate. Evolution toward Fourier Domain OCT (FD-OCT) gave rise to the second-generation OCT, which eliminated the need to mechanically scan the reference arm. These newer devices can be classified into either Swept Source (SS-OCT) or Spectral Domain (SD-OCT), as illustrated in Figure 1. Both methods are equivalent from a theoretical point of view. However, SD-OCT uses a broadband light source with a spectrometer at the interferometer exit. SS-OCT uses a laser with a narrow instantaneous line width that is rapidly tunable over a large wavenumber range, which is combined with a single detector [5]. Both SD-OCT and SS-OCT are capable of real-time imaging with resolution (5–10 μm), which is about two orders of magnitude finer than ultrasound imaging (0.2–2 mm) [6]. Detailed mathematical descriptions of OCT fundamentals can be found at [7,8].

Another modern OCT technology is the polarization-sensitive optical coherence tomography (PS-OCT). This is a set of hardware and software extensions to OCT that allows measuring the birefringence of local regions of the tissue [10]. A tissue is said to be birefringent if the real part of its refractive index is polarization state dependent. Tissues such as muscle, cartilage and tendons exhibit birefringence due to their internal arrangement of sub-cellular fibrous structures. When the tissue is damaged or undergoes necrosis, this structure degenerates with a corresponding reduction in birefringence. The degree of birefringence, thus, gives an indication of the degree of tissue damage [8,10].

Despite its capabilities and different imaging modalities, standard OCT systems cannot image internal organs due to their limited imaging depth. Therefore, endoscopic OCT systems were developed to enable OCT imaging inside the body. This technology complements the traditional endoscopic imaging systems, which are extensively used in clinical practice both for the diagnosis and treatment of diseases, but which are still limited by the lack of technologies able to assist surgeons in the detection of lesions and assessment of their true extent both superficially and in depth.

In this context, intraoperative OCT can provide important benefits given its capability to acquire high-resolution images of superficial tissue volumes with high contrast, which can help clinicians better estimate tumor margins. In addition, it has great potential to enable higher surgical precision based on image-guided maneuvers and real-time feedback on interactions between surgical instruments and the underlying tissue [11].

More specifically, this is the case of OCT-guided laser surgery, which is expected to offer additional benefits such as increased controllability and precision in tool–tissue interactions. This combination of OCT imaging and laser surgery can enable the achievement of precise surgical margins while contributing to prevent collateral damages, such as damage to blood vessels, nervous tissues, or other critical structures. Therefore, endoscopic OCT and OCT-guided laser surgery are active topics of research and technological development.

A number of reviews on endoscopic OCT imaging devices can be found in the literature. However, the most recent review paper on this topic was published by Gora et al. in 2017 [12]. In addition, OCT-guided laser surgery was covered only briefly in another review paper from 2017 [13]. Therefore, this paper aims to fill this gap in the literature, providing a concise and comprehensive updated summary of potentially enabling technologies for OCT-guided endoscopic laser surgery.

Specifically, this paper reviews the newest endoscopic OCT technologies and OCT-guided laser surgery systems, highlighting technological challenges and potential solutions. For this review, we used both the PubMed database and Google Scholar to search for recent literature. A total of 118 papers were collected with this search. Then, after filtering redundancies, 12 papers were finally selected as a representative subset covering all of the proposed technologies. Additionally, another 28 papers were identified through citations and included in this review.

The remainder of this paper is organized in three main sections. First, endoscopic OCT technologies are reviewed considering different technical characteristics, such as scanning geometry, actuator configuration, and type of actuators. Here, implementation challenges and proposed solutions are presented. Then, the subsequent section reviews the state of the art in OCT-guided laser surgery systems. Three major methods are identified for implementing such systems, including those using double-clad fiber (DCF), dichroic mirror or separate optical paths. Finally, the last section concludes the paper with a discussion on the open challenges and potential approaches to advance this new type of surgical technology toward clinical use.

## 2. Endoscopic OCT Technologies

Different endoscopic OCT (endoOCT) systems have been developed in recent years to acquire high-definition images of tissue volumes from internal organs. In these systems, the OCT sample arm is typically the only part that goes inside the patient, while the rest (i.e., the reference arm, laser source, and detector) are outside of the patient. This section highlights the challenges and reviews solutions proposed for implementing endoOCT systems, including the definition of scanning geometry, actuator type and actuator configuration for specific clinical applications.

### 2.1. Scanning Geometry

**Figure 2:**
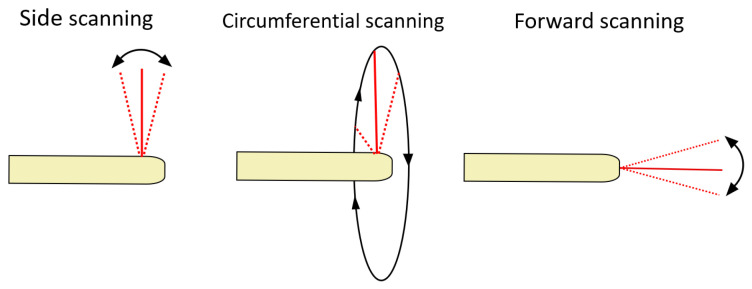
Scanning geometries of endoscopic OCT systems: side, circumferential and forward scanning.

In an endoOCT probe, laser scanners are required to perform the B-scan and C-scan. The challenge here lies in implementing such a scanner in a miniature form factor that is at the same time robust and compatible with the intended clinical application. In fact, depending on the application, the endoOCT scanning geometry may be circumferential, side-looking or forward looking. This characteristic allows classifying endoOCT probes as side scanning, forward scanning or circumferential scanning, as illustrated in Figure 2.

While a forward-scanning device may have a smaller field of view than a circumferential scanner, it can obtain images from the area in front of the probe. Therefore, forward-viewing OCT systems can be well suited for image guidance during biopsies, device placement, or treatments in which a sufficient space between the OCT probe and the sample surface is needed [12]. Consequently, they may be better suited for organs such as the larynx, prostate, ovaries, etc. A key challenge here is linked to miniaturization and the need to realize a scanning device with small outer diameter. This has been addressed by the development of fiber scanners, as demonstrated by the forward-scanning OCT probes created by Zhang et al. [14] and by Huo et al. [15].

Another challenge for the creation of forward-scanning probes regards the working distance of the endoOCT system, which can vary depending on the patient’s anatomy and the positioning of the probe. This variation has to be compensated for proper imaging, and this involves adjusting either the system’s working distance or the length of the OCT’s reference arm. A solution to this issue was proposed by Guo et al., who developed a rigid OCT probe with motorized working distance adjustment for in vivo human larynx imaging [16]. In addition, in a subsequent generation of the system, a long GRIN lens was used to address this same issue [17].

**Figure 3:**
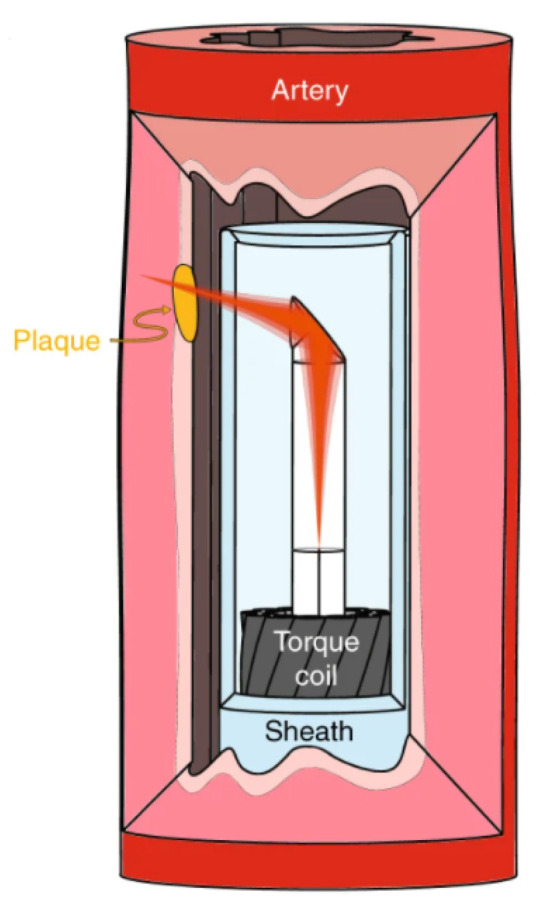
Schematic of a 3D-printed OCT endoscope inside an artery by [18]. Figure licensed under CC BY 4.0.

Circumferential scanning OCT probes have an ideal imaging geometry for circular lumens and afford significant miniaturization. These characteristics make them ideal for intravascular OCT imaging, as demonstrated by the ultrathin probe (0.457 mm) developed by Li et al. and illustrated in Figure 3 [18]. However, they can only image perpendicular to the probe and cannot image structures or interventional procedures occurring out of this plane [19]. From an engineering point of view, circumferential scanning tends to be less challenging than forward scanning because it does not require a distal beam scanner [12]. Nonetheless, circumferential scanning systems have their own challenges, such as the creation of image volumes (C-scans) and obtaining proper coverage of the tissue volume of interest.

Typically, e.g., in endovascular and gastroenterology applications, C-scans are obtained by circumferential scanning probes through a pullback motion during laser scanning, after it is initially inserted into to lumen of interest. This retraction can be performed manually, such as in tethered capsule OCT probes [20,21,22], or in a motorized fashion using a proximal control stage, as in the endomicroscopy systems developed by Abbott [23] and NinePoint Medical, Inc. [24].

As far as guaranteeing proper coverage, which is a particular challenge in gastroenterology due to the collapsible nature of the esophagus, balloon-based OCT catheters have been developed [25]. This solution was shown to effectively center and stabilize the catheter within the esophagus, enabling it to maintain an optimal focal distance along the entire circumference of the esophageal wall. Currently, balloon-based OCT catheters are regularly used for volumetric laser endomicroscopy (VLE).

Other challenges of circumferential scanning OCT probes include optical aberrations, shadow artifacts, Non-Uniform Rotational Distortions (NURD), and difficulties in co-localizing subsequent biopsy with the OCT images. To address the issues regarding chromatic and astigmatic aberrations, Yuan et al. developed an OCT probe with a ball lens and a multi-mode fiber (MMF)-based spacer instead of standard GRIN lens-based probes [26]. Li et al., on the other hand, proposed the use of diffractive optics to correct chromatic aberrations [27].

**Figure 4:**
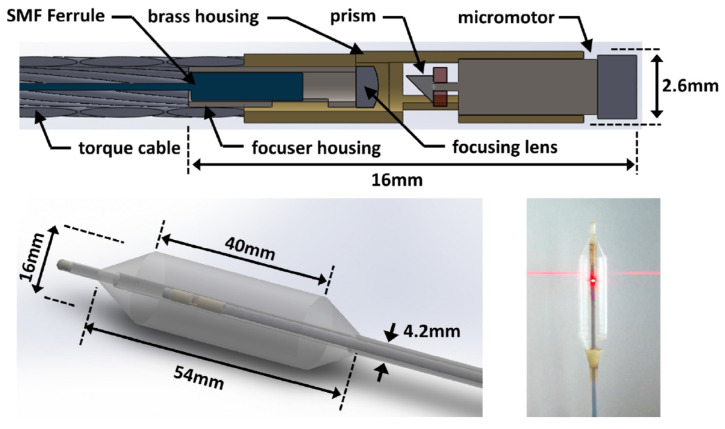
Example of a micromotor-based balloon catheter for OCT imaging, used with permission from [28] © The Optical Society.

The typical shadow artifact of circumferential scanning probes was avoided by López-Marín et al. with the development of a scanning system in which the OCT light beam goes around the motor [22]. NURD has been addressed both with distal scanning mechanisms, such as in the system developed by Lee et al. [28] (Figure 4), and through software, such as the feature-based tracking technique proposed by Lo et al. [29]. Finally, the co-localization of biopsy with the OCT images has been successfully addressed through high-power laser marking of the tissue, as demonstrated by Liang et al. in tethered capsule endomicroscopy [30] and used in the commercial ballon-based NvisionVLE Imaging System [24].

**Figure 5:**
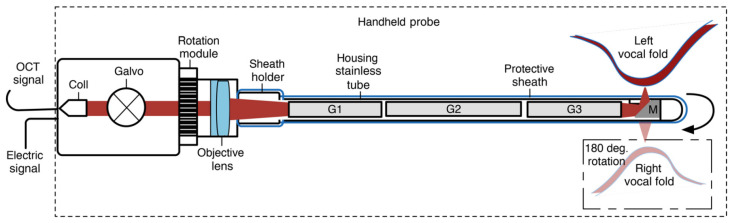
Schematic diagram of an OCT imaging system for pediatric laryngoscopy with a proximal scanning mechanism by [32]. Figure licensed under CC BY 4.0.

Side-scanning OCT probes offer potential to acquire 3D endoscopic OCT images without NURD and without the need for a pullback motion. However, with respect to their circumferential-scanning counterparts, implementation is more complex and miniaturization is more challenging. Nonetheless, effective side-scanning OCT probes have been developed both based on MEMS technology at the distal tip, such as the device presented by Kim et al. [31], and based on proximal beam steering by mirror-mounted galvanometers, as demonstrated by Benboujja et al. [32] (Figure 5).

### 2.2. Actuator Configuration

The laser scanner must be actuated to acquire B-scan and C-scan images. This can be completed using either proximal or distal actuators. Proximal scanning OCT endoscopes usually provide circumferential scanning, but forward scanning is also possible, as reported by Xie et al. [33]. Given the external position of the actuators, these devices typically have a smaller diameter and are simpler than distal scanning probes. This makes them suitable for lumen organs such as the vasculature, esophagus, etc. However, proximal scanning probes tend to suffer from NURD, and they usually cannot image outside the perpendicular plane due to their circumferential scanning nature.

**Figure 6:**
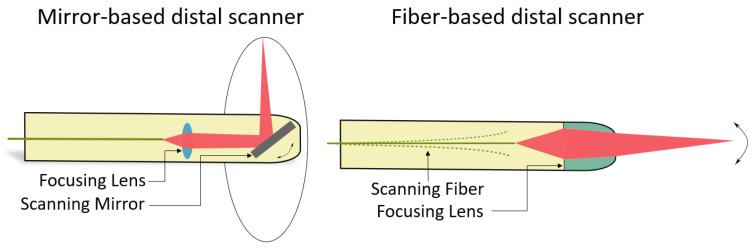
Examples of distal scanning endoscopic OCT probes based on a mirror and on a fiber scanner.

Distal scanning OCT endoscopes can be achieved based on the manipulation of a mirror, a lens, or an optical fiber. Fiber-based scanners are typically forward scanning, whereas mirror-based systems are usually side viewing, as illustrated in Figure 6.

### 2.3. Types of Actuators

**Figure 7:**
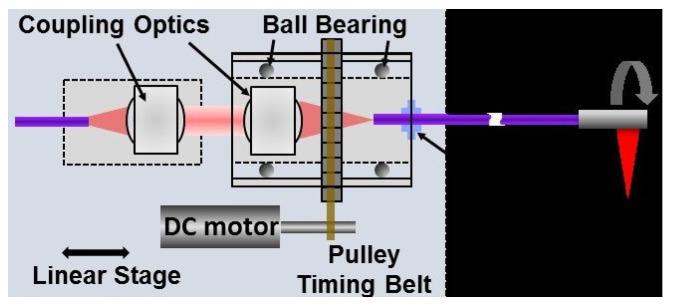
Proximal scanning system for an endoscopic OCT. Light from a stationary source fiber is coupled to a rotating endoscopic probe, used with permission from [12] © The Optical Society.

Endoscopic OCT scanners have been achieved using a wide range of technologies, including MEMS-mirrors, MEMS-lens, micro-motor, galvanometer mirrors, wedge lenses, fiber scanners and others. Proximal scanning probes are usually based on a rotating endoscope. A rotary joint is used to couple light from a stationary source fiber to the rotating endoscope. This can be achieved with a pair of air-coupled lenses. The first one collimates the light from the source fiber and the second one focuses the collimated beam into a single mode fiber (SMF) used in the endoscopic probe. The first lens is normally fixed in space, while the second one is mounted within a ball bearing that can be rotated by a standard motor, as shown in Figure 7.

Distal scanning probes can be actuated by different actuator types, such as electrostatic, electromagnetic, piezoelectric, electrothermal and micro-motor. Based on recent reviews, including [2,34,35,36], the pros and cons of these actuation technologies can be summarized as follows: (1) Electrostatic actuators have low power consumption and can achieve fast scanning but require high driving voltages. (2) Electromagnetic actuation can achieve large optical scan angles with low driving voltage, but they might cause electromagnetic interference (EMI) issues in other medical devices. (3) Piezoelectric actuation is fast and low power, but it presents coupling non-uniformity and hysteresis, is affected by charge leakage, and requires high voltage for actuation. (4) Electrothermal actuation combines the advantages of large optical scan range, low drive voltage, no electrostatic discharging problems, no EMI issue, and good scan linearity. However, they present cooling issues and residual bending stress that limit the actuation speed. (5) Micro-motor distal actuation can minimize NURD and achieve a faster imaging speed than proximal scanning, but the application of such actuators is limited both by their cost and potential for miniaturization [12].

## 3. OCT-Guided Laser Surgery

In addition to imaging, lasers also offer several benefits for surgery, such as non-contact tissue ablation, precise cutting, hemostasis, low cicatrization, reduced postoperative pain, and reduced tissue swelling. In addition, they can be combined with endoscopes, microscopes, and surgical robotic systems. However, laser surgery is challenging because of the complex laser–tissue interaction process. This leads to poor ablation depth control and tissue specificity, increasing the risk of accidental damage to nerves or blood vessels [37].

Intraoperative imaging can be a key assistive technology to improve surgical margins definition and to help prevent damage to critical structures in the operating field. In fact, the current lack of intraoperative imaging capabilities has been associated to increased chances of over or under-treatment [38]. Under-treatment requires repeated surgery with the associated cost, while over-treatment can lead to complications and reduce surgical quality.

Although various intraoperative imaging technologies such as MRI, CT and ultrasound are available, OCT offers distinct advantages for being non-contact, low cost, radiation-free, and fiber-based. In addition, as discussed in the previous section, OCT can be integrated with hand-held probes, laparoscopes, catheters, and endoscopes. Therefore, OCT can be an optimal technology for intraoperative imaging and guidance during delicate operations such as precise laser surgeries.

In this section, the state-of-the art in OCT systems for guiding laser surgery is reviewed. The references are organized according to the methods used to combine a high-power surgical laser with OCT. To date, these methods include: (1) double-clad fiber [39]; (2) dichroic mirror [40]; and (3) separate optical paths [41].

### 3.1. Double-Clad Fiber (DCF)

**Figure 8:**
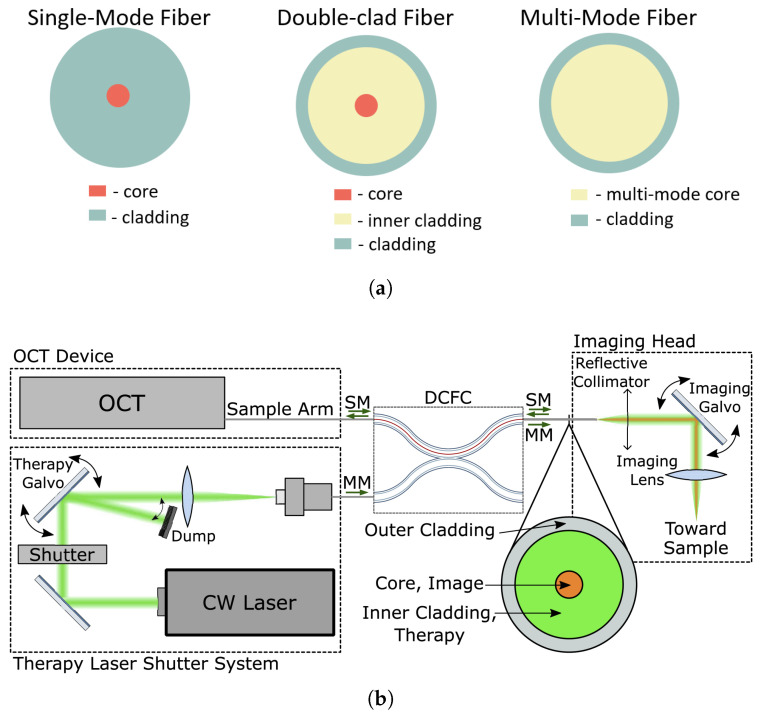
OCT-guided laser surgery systems based on double-clad fibers. (**a**) Different types of optical fibers: single-mode fiber (SMF), double-clad fiber (DCF) and multi-mode fiber (MMF). (**b**) Experimental setup proposed for co-localized OCT imaging and laser therapy, used with permission from [38] © The Optical Society.

When light travels in an optical fiber, only waves at certain discrete angles greater than or equal to the fiber’s critical angle will propagate, resulting in discrete modes or eigenfunctions. In a single-mode fiber (SMF), due to the small core diameter, only one mode of light propagates through it. Whereas in the multi-mode fiber (MMF), multiple modes propagate. Double-clad fibers (DCF) have a single-mode core surrounded by a multi-mode inner cladding, as shown in Figure 8a. The inner core is used for OCT imaging, and the outer core is used for laser therapy. DCF can support wavelengths up to 1750 nm [38]. However, the DCF is known to have cross-talk between the single-mode core and inner cladding, which results in ghost images and/or increased noise floor [42].

Maltais-Tariant et al. synchronized the therapy galvanometer and imaging galvanometer of a double-clad fiber system to deliver the treatment laser only in targeted areas [38]. A shutter was used to stop the therapy laser once the targeted therapy duration or coagulation was reached. A diagram of the system setup is presented in Figure 8b. It included a reflective collimator based on a 90° off-axis parabolic mirror, which contributed to avoid damaging the OCT system with the therapy laser’s high power. Ghost artifacts induced by the DCF were reduced by splicing an extra 1 m of Single Mode Fiber (SMF) between OCT and DCF. The process was demonstrated on an ex vivo rat tongue and abdominal muscles for ablation depths ranging from 500 to 1000 µm.

Jivraj et al. coupled an OCT signal into the signal core of a fiber laser using a commercially available pump combiner [43]. The tilted fiber gratings were used to protect the OCT system from the high-power laser. The saturation of the OCT’s balance detector by the reflected leakage light from the combiner was avoided by firing ablation pulses during the latter half of the A-line period, when no OCT data are being gathered. The system demonstrated the feasibility of the method, but the following drawbacks were observed: (1) OCT sample beam dispersion due to the fiber laser; (2) attenuation of OCT due to the large mode area of the fiber laser; and (3) bending loss. In addition, the system did not incorporated any steering control of the end of the fiber.

Chang et al. described the use of en-face OCT maps to classify tumors and non-tumor tissues based on Otsu’s method [44]. Then, by combining this information with laser ablation units, they generated driving signals for the therapy laser. In the system, the use of a DCF avoided the need for image registration. However, the filters used to avoid high-power backscattered radiation attenuated the OCT signal, affecting image quality. An additional limitation included the fact that the system did not consider potential patient motions between the acquisition of OCT images and the subsequent laser therapy.

### 3.2. Dichroic Mirror (DM)

A dichroic mirror allows light of a certain wavelength to pass through, while light of other wavelengths is reflected. They are made by alternating layers of optical coatings with different refractive indices. The interfaces between these layers produce phased reflections, selectively reinforcing certain wavelengths of light. Reflective collimators are typically based on a 90° off-axis parabolic mirror, which has a focal length that remains constant over a broad wavelength range. In the studies reviewed in this section, dichroic mirrors and reflective collimators are used to align OCT and high-power lasers. This combination of optics is useful to avoid damaging the OCT setup with the intense backscattered high power laser [45].

**Figure 9:**
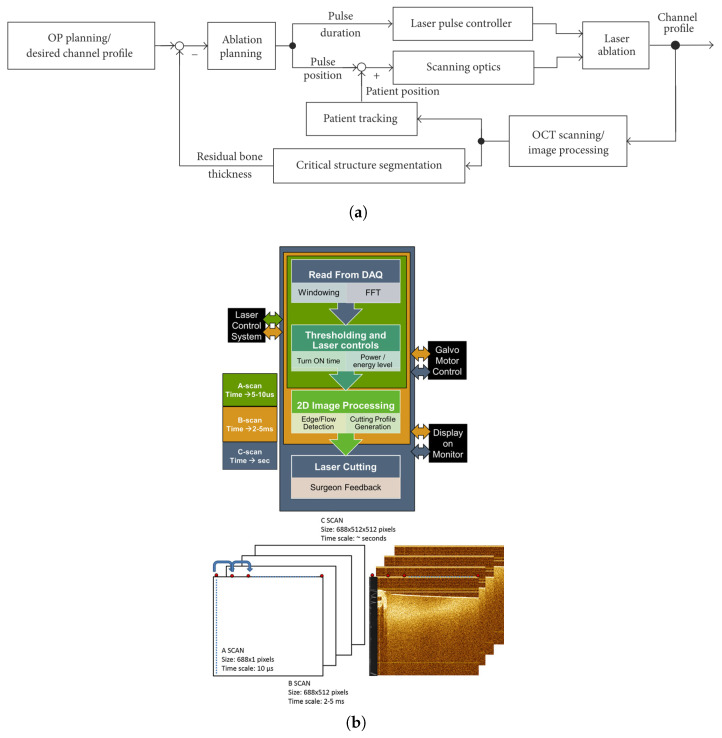
OCT-guided laser surgery systems based on dichroic mirrors. (**a**) Control loop scheme of the OCT-guided laser cochleostomy by [46] is licensed under CC BY 3.0. (**b**) Data flow and representation in the smart laser surgical system, used with permission from [40] © The Wiley.

Zhang et al. investigated the potential of OCT-guided laser surgery in cochleostomy (inner ear surgery) [46]. This procedure involves drilling the cochlea while avoiding damage to the cochlear endosteum. However, due to the small diameter and thickness of the endosteal layer, the drilling process reaches the limits of human capabilities. Thus, the researchers used OCT images to control the pulse position and duration of a high-power laser in closed-loop, as illustrated in Figure 9. This enhanced the precision of the operation, preventing laser exposure to the critical structure. Nonetheless, detection of the critical structure was difficult due to the presence of the highly scattering bone. Therefore, they developed a speckle averaging technique called History Compounding. Finally, they also used OCT as an accurate optical tracking system by locating small laser-ablated landmarks surrounding the cochleostomy. However, open challenges for clinical translation were still identified. For example, the experiments demonstrated that 100% protection of the endosteum was not guaranteed, as the channel bottom penetrated the “stop surface” in some points. In addition, the critical structure segmentation was semi-automatic and OCT imaging, processing, and CO${}_{2}$ laser control were conducted by three independent software packages. This resulted in high operation complexity, which may lead to increased surgical time.

Katta et al. used prior OCT images to obtain a laser ablation pattern avoiding blood vessels in a tissue phantom [40]. As shown in Figure 9, the laser power and its ON/OFF times were dynamically controlled based on a “blow-off” model during A-scan OCT. However, the field curvature in the scanning element and the laser’s finite ON/OFF time resulted in an uneven cut. Recently, they used prior OCT images to compute the three-dimensional tumor margin and angiography images to guide the coagulation and ablation steps in a brain cancer surgery of a xenograft model [47].

In 1999, Boppart et al. used OCT to image 65 sites on five ex vivo rat organ tissues before, during, and after laser ablation [48]. Following imaging, tissue registration was achieved by histologic processing to confirm the ablation site’s morphology. They identified that the carbonized tissue layer rapidly absorbs and scatters both the incident argon laser and OCT imaging beams. Because of this, OCT imaging penetration is reduced and shows shadowing artifacts. The same group performed another study on prostate ablation [49]. In this case, it was noted that the loss of cell viability in the in vitro specimens reduced the contrast when compared to in vivo studies. It was also suggested that the presence of blood will further reduce OCT imaging penetration.

Leung et al. ablated ex vivo cortical (outer bone) using pulsed lasers while imaging in real time [50]. The intense backscattered light from the ablation process was avoided using inline coherence imaging (ICI). The ICI is analogous to M-mode (‘‘motion-mode’’) ultrasonography. This provided the depth information in real time, avoiding tomography. They studied both the thermal and the ultrashort pulsed regimes of the ablation laser, finding that carbonization from the thermal regime affected image quality.

Ohmi et al. studied tissue laser ablation using OCT [51]. For this, they controlled the ablation lasers using an electronic shutter and used OCT to image the ablation crater. Laser ablation and OCT imaging were repeated following a sequential automatic procedure. Using this method, they performed experiments on human teeth and chicken bones. The depth of the crater and the ablation rate was determined from the OCT images. The ablation rate was estimated as 0.21 μm per pulse on the human tooth. However, the resolution of the OCT imaging system was only 10 μm, leading to the imprecise measurement. Real-time feedback was not possible due to the slower data acquisition of TD-OCT used.

### 3.3. Separate Optical Paths (SOP)

Fan et al. integrated a bench-top OCT setup and an endoscope systems with fiber laser to create a proof-of-concept system for OCT diagnostics and guidance during laser surgery [41]. In the system, the OCT beam and the ablation laser have separate optical paths, but they are manually aligned using the endoscopic images. In this setup, OCT is initially used to generate a tumor map and then to monitor the laser ablation. The results demonstrated the potential of OCT-guided laser surgery for accurate tumor resections. Nonetheless, significant challenges remained open to achieve full integration of OCT and laser ablation into an endoscopic system.

**Figure 10:**
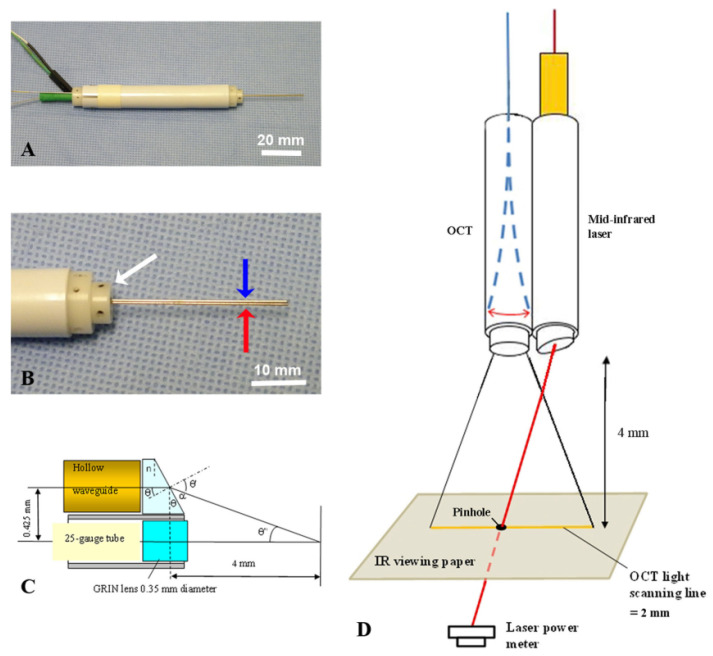
Combined miniature B-scan OCT and surgical laser in an intraocular probe, used with permission from [52] © The Wiley. (**A**) Picture of the device. (**B**) Magnified picture showing the support strucutre (white arrow) and the combined OCT (red arrow) and laser (blue arrow) probe tip. (**C**) Details of the optical components that allow co-planar ablation and imaging. (**D**) Diagram of the combined OCT and laser probe tip showing the scanning optical fiber.

Li et al. fastened an electromagnetically actuated forward scanning OCT probe of 0.5 mm diameter with a hollow waveguide to permit co-planar ablation and imaging [52]. The waveguide’s tip had a CaF_2_ window to align the laser with the OCT signal, as shown in Figure 10. The system was tested in gelatin and ocular tissues. Real-time OCT images were used to monitor the lasing operation to avoid ablating critical structures. The combined probe functioned as long as the surface being ablated was within the scanned volume, approximately 3 to 5 mm from the probe tip.

## 4. Conclusions

This paper reviewed the state of the art in endoscopic OCT systems and the recent advances toward OCT-guided endoscopic laser surgery. Most of the endoscopic OCT systems developed to date provide circumferential scanning and are based on proximal actuation. These systems are designed to address the challenges of specific clinical applications, which include endovascular surgery and gastroenterology as the current main drivers for the development of the technology. Nonetheless, other endoscopic OCT configurations (such as forward scanning probes) and other promising application areas (such as in laryngology and neurosurgery) are being explored. Future work will likely expand research in these directions, enhancing the scope of endoscopic OCT systems.

Regarding OCT-guided laser surgery, it is clear from this review that three major factors should be considered when designing such systems. First, the OCT detector should not be saturated with the backscatter reflected light from the ablation laser. This can be achieved by using a filter or by synchronizing the timing of the OCT imaging and the therapy laser pulse, as described in the papers reviewed herein. Second, the focusing optics have to be carefully designed considering both lasers. Mirrors are a good choice as they provide broadband and high thermal load. However, they make the integration into an endoscopic system more challenging. Nonetheless, the use of MEMS technology may be a viable solution to these miniaturization and integration challenges. Finally, the three major methods to co-align the OCT laser and a therapy laser include the use of a double-clad fiber (DCF), a dichroic mirror, or separate optical paths. Dichroic mirrors are robust and simple, but their incorporation into an endoscopic device is challenging given the miniaturization constraints. Therefore, this method tends to be more suitable for microscope or exoscope-based devices. On the other hand, DCF allows easier integration with an endoscopic device, but the OCT imaging quality is reduced, as it is affected by cross-talk and increased levels of noise. Further research would be needed to address these issues. Finally, the use of separated optical paths, such as two parallel optical fibers, is a promising method to combine OCT and a high-power laser given its simplicity and significant potential for miniaturization.

A summary of the main systems developed to date for OCT-guided laser surgery is presented in Table 1. These systems show good progress toward this new type of surgical technology, but none of them could yet demonstrate endoscopic operation. Further research is needed to optimize systems in that direction and satisfy clinical constraints, which include, for example, miniaturization for endoscopic system integration, compatibility with sterilization, and deployment of intuitive user interfaces for high usability and efficient control.

Additional open challenges toward clinically viable OCT-guided endoscopic laser surgery systems include the development of robust methods to couple OCT and high-power lasers that are able to provide top OCT image quality; the implementation of efficient methods for controlling laser parameters in real time to achieve the ablation of precise tissue volumes; the development of reliable methods for tissue tracking and motion compensation; and the creation of efficient methods and mechatronic systems for positioning the surgical system at an optimal distance and angle with respect to the therapy area.

Addressing all these challenges will require significant amounts of research and development in upcoming years. For instance, the method to couple OCT and high-power lasers has to be consolidated and refined to address the needs of specific clinical applications. The pairing of laser–tissue interaction modeling and cognitive systems may be investigated to enable effective control over ablation volumes. AI and Deep Learning methods may be researched as potential solutions to the tissue-tracking challenges. Finally, robotic systems, such as robotic catheters or robotic endoscopes, may be needed for addressing the positioning and control challenges of OCT-guided endoscopic laser surgery systems.

Although the road toward clinical systems may still be long, the results achieved to date strongly support further investment in the research and development of OCT-guided endoscopic laser surgery systems. There is general agreement that this new theranostics technology can bring important benefits for surgical applications, especially for precision surgeries in delicate human organs. These include the potential for more precise surgical margins in oncological applications and safer procedures with better preservation of healthy tissues and critical structures.

## Figures and Tables

**Table 1:** Summary of main systems proposed to date for OCT-guided laser surgery. None of these systems could demonstrate endoscopic operation.

Ref.	OCT Type	Laser Wavelength	Laser Power	Config.
[38]	SS-OCT	532 nm	10 W	DCF
[43]	SS-OCT	1064 nm	3.35 W	DCF
[44]	SD-OCT	1064 nm	1–100 W	DCF
[46]	SS-OCT	10.6 μm	4.2 to 28.5 mJ/pulse	DM
[40]	SS-OCT	1940 nm	15 W	DM
[49]	TD-OCT	514 nm	2 w	DM
[51]	TD-OCT	1.06 μm	N/A	DM
[41]	SD-OCT	1.06 μm	75 W	SOP
[52]	SD-OCT	6.1 μm	0.7 mJ/pulse	SOP

## Data Availability

Data sharing not applicable.
